# Cancer Risks for Relatives of Children with Cancer

**DOI:** 10.1155/2014/806076

**Published:** 2014-03-27

**Authors:** John A. Heath, Elizabeth Smibert, Elizabeth M. Algar, Gillian S. Dite, John L. Hopper

**Affiliations:** ^1^Children's Cancer Centre, Royal Children's Hospital, Flemington Road, Parkville, VIC 3052, Australia; ^2^Centre for Molecular, Environmental, Genetic and Analytic Epidemiology, School of Population and Global Health, University of Melbourne, Parkville, VIC 3053, Australia; ^3^Murdoch Children's Research Institute, Parkville, VIC 3052, Australia

## Abstract

We determined the extent and distribution of cancers in relatives of 379 children newly diagnosed with cancer. Family history was collected from 1,337 first-degree and 3,399 second-degree relatives and incidence compared with national age- and gender-specific rates. Overall, 14 children (3.7%) had a relative with a history of childhood cancer and 26 children (6.9%) had a first-degree relative with a history of cancer, with only one of these having an identifiable familial cancer syndrome. There was a higher than expected incidence of childhood cancer among first-degree relatives (parents and siblings) (standardized incidence ratio (SIR) 1.43; 95% CI 0.54–5.08). There was also a higher than expected incidence of adult cancers among first-degree relatives (SIR 1.45; 95% CI 0.93–2.21), particularly in females (SIR 1.82; 95% CI 1.26–3.39). The increased family cancer history in first-degree females was largely attributable to an effect in mothers (SIR 1.78; 95% CI 1.27–3.33). The gender-specific association was reflected in higher than expected incidence rates of breast cancer in both mothers (SIR 1.92; 95% CI 0.72–6.83) and aunts (SIR 1.64; 95% CI 0.98–2.94). These findings support the hypothesis that previously undetected familial cancer syndromes contribute to childhood cancer.

## 1. Introduction

Childhood cancer is an important component of the total cancer burden worldwide. Survival rates continue to improve with the advent of more refined treatments and better supportive care. Today over 80% of children with cancer are alive for at least five years, and the majority of these are cured [1]. As a consequence, many sufferers of childhood cancer are now living into adulthood and having families of their own. It has been estimated that about 1 in 900 adults aged 18 to 44 years is a cancer survivor [2]. Among this group, those with an inherited susceptibility to cancer will transmit their genetic fault to a proportion of their children. They are also at risk of developing a second cancer during their adult life [3].

While the causes of the majority of childhood cancers are largely unknown, there are a number of clinical syndromes for which the evidence for an excess cancer risk in children is most persuasive. These include Li-Fraumeni syndrome, neurofibromatosis type I, inherited retinoblastoma mutations, familial Wilms tumor, and some disorders of DNA repair [4]. These syndromes collectively account for less than 10% of all childhood cancers, however. Beyond this, it is not clear if cancer in childhood increases the risk of cancer in relatives. A number of recent studies have described an association between childhood cancer and cancer in first-degree relatives [5–7], particularly siblings [5, 7] and mothers [5]. In this study, we describe the distribution of cancers in Australian children and estimate the risks of cancer in their relatives.

## 2. Materials and Methods

### 2.1. The Victorian Paediatric Cancer Family Study (VPCFS)

The VPCFS is a cohort of newly diagnosed childhood cancer patients. It was established from the clinical services at the Royal Children's Hospital (RCH) and the Monash Medical Centre (MMC), between which over 95% of children under 10 years of age and 83% of children aged 10–15 years with cancer within the State of Victoria, Australia, are treated [8]. Children were eligible for inclusion if they had a diagnosis of any cancer before age 15 and when initial treatment was given at one of the participating centres between October 1998 and October 2002. The Human Research Ethics Committee at each participating institution reviewed and approved the protocol before enrolment commenced.

A total of 486 (422 at RCH and 64 at MMC) incident cases of childhood cancer were diagnosed during the study period. Of these, 21 families were found to be ineligible (seven non-English speaking parents, seven where the proband was aged over 15 years, six where the proband did not have their initial treatment at either service, and one where the proband was adopted). Of the remaining 465 families, 21 were not contactable, 53 refused to participate, 6 were lost to followup, and 6 later withdrew consent. This left 379 (82%) families participating (340 at RCH and 39 at MMC), from which we interviewed 351 (92.6%) fathers and 371 (97.9%) mothers. In 343 (90.5%) families, both the mother and father were interviewed, while in 28 (7.4%) families only the mother was interviewed and in 8 (2.1%) families only the father was interviewed.

### 2.2. Family History of Cancer

At the time of enrolment, both parents of the affected child were asked a baseline questionnaire that included information on lifestyle and environmental risk factors and a detailed family history of cancer. Where half siblings were identified, the appropriate, biologically relevant family history was obtained. The family history of cancer questionnaire asked about all of the affected child's siblings, parents, aunts, uncles, and grandparents (i.e., all first-degree and second-degree relatives). Parents were asked to identify each first-degree and second-degree relative, any known cancer diagnoses with the age (in years) at diagnosis, and type of malignancy, together with their current age or age at the time of death. The information collected was reviewed and classified by the authors with expertise in cancer (JAH; ES). The World Health Organization's International Classification of Diseases for Oncology, 3rd Edition (ID-O-3) scheme, was used for classification of site of malignancy [9]. Nonmelanoma skin cancers, nonmalignant tumours, and* in situ* cancerswere not included in the analyses.

To address the potential contribution of known familial cancer syndromes, we examined the medical records of the 26 affected children who had a first-degree relative with cancer. In particular, we sought clinical evidence for Li-Fraumeni syndrome, neurofibromatosis type 1, familial retinoblastoma, and familial Wilm's tumour syndrome.

### 2.3. Statistics

Person-years at risk for the cohort of relatives were calculated as the time to date of diagnosis of cancer, date of death, or date of interview completion, whichever occurred earliest. Australian population-based cancer incidence data, specific for gender, age, and year of birth, were obtained from the Australian Institute of Health and Welfare [10]. Standardised incidence ratios were used to compare the number of observed cancers in relatives with the number expected in the Australian population. Robust estimates for confidence intervals were calculated to account for potential clustering within a family. Statistical analyses were conducted with Stata Version 12 [11]. All statistical tests were two-sided and a *P* value <0.05 was considered statistically significant.

## 3. Results

The distribution of childhood cancers diagnosed in the cohort of 379 children is summarized in Table 1. A family history for cancer was identified in 4,736 (1,337 first-degree and 3,399 second-degree) relatives with a total of 211,394 person-years of followup. Fourteen of the 379 (3.7%) families reported a positive history for childhood cancer in any relative, with none having more than one case identified. Twenty-six children with cancer (6.9%) had a first-degree relative (parent or sibling) with a history of cancer.

The results for the family history of cancer in all childhood cancer patients are summarized in Table 2. There was a higher than expected, though not statistically significant, incidence of childhood cancer among first-degree relatives (SIR 1.43; 95% CI 0.54–5.08). There was also a higher than expected, though not statistically significant, incidence of cancer among first-degree relatives (SIR 1.45; 95% CI 0.93–2.1). There was a statistically significant increase in cancer among female first-degree relatives (SIR 1.82; 95% CI 1.26–3.39). The increased family cancer history in first-degree females was largely attributable to an effect in mothers (SIR 1.78; 95% CI 1.27–3.33) but was also observed in sisters (SIR 2.15; *n* = 1). Elevated cancer incidence rates were also observed in aunts (SIR 1.48; 95% CI 1.11–2.00). The gender-specific association was reflected in higher than expected incidence rates of breast cancer in mothers (SIR 1.92; 95% CI 0.72–6.83), aunts (SIR 1.64; 95% CI 0.98–2.94) and to a lesser extent grandmothers (SIR 1.04; 95% CI 0.78–1.40). Although numbers were small, an increased cancer incidence in relatives appeared to be most apparent in the children with sarcomas (SIR 2.58; 95% CI 0.99–8.52), embryonal tumors (SIR 2.12; 95% CI 0.71–9.34), and brain tumors (SIR 1.52; 95% CI 0.70–3.92).

Of the 26 families with a history of cancer within a first-degree relative, only one met the criteria for a clinically recognizable familial cancer syndrome (Li Fraumeni syndrome). The increased rates of cancer in female first-degree relatives (SIR 1.68; 95% CI 1.03–2.93), including mothers (SIR 1.78; 95% CI 1.09–3.09) and aunts (SIR 1.49; 95% CI 1.12–2.02), remained when this child and family were removed from the analyses.

## 4. Discussion

Our small, population-based study suggests an increased risk of childhood cancer and some adult cancers among relatives of childhood cancer patients that are not accounted for by clinically identifiable familial cancer syndromes. These findings were seen in both first- and second-degree relatives. Although some of our findings did not reach statistical significance, they are largely consistent with previously published unselected childhood cancer cohorts [5–7, 12] and add to a growing body of evidence that unidentified genetic risk exists in some families. In addition to the small size of the study, other limitations include the use of family history in isolation and the absence of some parental input to family history of cancer. While we were not able to cross-reference reported history of cancer with cancer registry data, a similar study in Sweden confirmed underreporting of cancer in relatives by history [6]. Furthermore, in the small minority of cases where both parents were not available to provide the relevant family history of cancer, underreporting of the family history of cancer is also likely. Allowing for these two study biases towards the null hypothesis is therefore likely to strengthen the positive findings we have reported. This underreporting may also explain why a large number of apparent protective effects occurred in second-degree relatives, where direct, personal access to a family history of cancer is not always possible.

Overall, first-degree relatives (siblings and parents) had an increased incidence of childhood tumors. This is consistent with the higher occurrence of childhood cancers among siblings demonstrated in a study of 51,000 children who developed cancer under the age of 15 in the UK, even after those with a genetic etiology were excluded [12]. It was also reported in a cohort of 13,703 childhood cancer survivors in North America (SIR 1.5; 95% CI 1.35–1.7) [7].

The apparent association of childhood cancer with adult cancers in female relatives is fascinating and supports a recently published population-based study from Utah, USA [5], where a higher risk of adult cancer was restricted to mothers and siblings (SIR 1.31; 95% CI 1.11–1.56) but was not observed in fathers [5]. It is interesting to note that the effect was greatest for children younger than 5 years at diagnosis (SIR 1.48; 95% CI 1.13–1.95), suggesting a strong ante- or perinatal effect. Factors which may be related to maternal effects on childhood cancer risk include maternal age of pregnancies, altered* in utero* exposures, and birth weight [13].

Our finding of an increased prevalence of breast cancer in female relatives also supports recently published Swedish data [6]. A number of related studies have also observed higher rates of breast cancer in mothers and sisters [14–16]. The potential link between breast cancer and childhood cancer is of great interest, given past reports of increased rates of childhood cancer in families carrying a BRCA1 or BRCA2 mutation [17, 18]. Alternative common genetic pathway alterations such as the IGF1 axis have also been postulated [6].

Our study was not designed nor powered to examine the relationship between specific childhood cancer types and family history of cancer, so it must be acknowledged that potential associations may have gone undetected. Certainly, others have postulated a link between family history of cancer and the more common subtypes, childhood leukaemia [19], childhood lymphomas [20], and childhood brain tumours [21].

The epidemiology of childhood cancer described here is the starting point for further explorations into identifying previously unrecognized genetic predisposition to childhood cancer. We are currently undertaking whole genome sequencing of these children with cancer and their affected first-degree relatives' germline DNA in order to identify the role of previously described and novel genetic mutations to account for our findings.

## Figures and Tables

**Table 1:** The distribution of childhood cancers enrolled in the Victorian Paediatric Cancer Family Study.

Cancer type	*N *	(%)
Leukemia	171	(45)
Acute lymphoblastic leukemia	145	
Acute myeloid leukemia	26	
Lymphoma	31	(8)
Hodgkins' lymphoma	15	
Non-Hodgkin's lymphoma	16	
Central nervous system	80	(21)
Glioma	43	
Medulloblastoma	14	
Germ cell	4	
Other	19	
Sarcoma	35	(9)
Bone—osteosarcoma	8	
Bone—Ewing's sarcoma	11	
Soft tissue sarcoma	16	
Neuroblastoma	17	(4)
Kidney	30	(8)
Liver	4	(1)
Retinoblastoma	2	(1)
Germ Cell—Non-CNS	9	(2)

Total	379	(100)

**Table 2:** Number of observed (O) and expected cancers (E) and standardised incidence ratios (SIR) and 95% confidence interval (95% CI) for cancer risk for relatives of proband.

Relationship to proband	O	E	SIR	95% CI
First-degree	28	19.8	1.45	0.98–2.21
Male	10	10.3	0.97	0.51–1.99
Female	18	8.9	2.00	1.26–3.39
Parents	25	18.7	1.36	0.93–2.08
Fathers	8	9.7	0.82	0.40–1.86
Mothers	17	8.4	2.00	1.27–3.33
Siblings	3	1.0	2.93	0.93–14.1
Second-degree	325	375.9	0.86	0.77–0.97
Male	163	208.4	0.78	0.67–0.91
Female	162	167.6	0.97	0.84–1.12
Grandparents	258	318.0	0.81	0.72–0.92
Grandfathers	144	183.2	0.79	0.67–0.92
Grandmothers	114	134.9	0.85	0.71–1.01
Uncles and Aunts	67	57.9	1.16	0.90–1.52
Uncles	19	25.2	0.75	0.46–1.32
Aunts	48	32.7	1.47	1.11–1.99
